# Biomarker kinetics in the prediction of VAP diagnosis: results from the BioVAP study

**DOI:** 10.1186/s13613-016-0134-8

**Published:** 2016-04-14

**Authors:** Pedro Póvoa, Ignacio Martin-Loeches, Paula Ramirez, Lieuwe D. Bos, Mariano Esperatti, Joana Silvestre, Gisela Gili, Gema Goma, Eugenio Berlanga, Mateu Espasa, Elsa Gonçalves, Antoni Torres, Antonio Artigas

**Affiliations:** Polyvalent Intensive Care Unit, Centro Hospitalar de Lisboa Ocidental, São Francisco Xavier Hospital, Estrada do Forte do Alto do Duque, 1449-005 Lisbon, Portugal; NOVA Medical School, CEDOC, New University of Lisbon, Lisbon, Portugal; Critical Care Center, Sabadell Hospital, Corporación Sanitaria Universitaria Parc Taulí, Universitat Autonoma de Barcelona, Sabadell, Spain; CIBER de Enfermedades Respiratorias (CIBERES), Madrid, Spain; Intensive Care Unit, University Hospital La Fe, Valencia, Spain; Department of Intensive Care, Academic Medical Center, University of Amsterdam, Amsterdam, The Netherlands; Respiratory Disease Department, Hospital Clínic i Provincial de Barcelona, IDIBAPS, Barcelona, Spain; Laboratory Department, UDIAT, Corporación Sanitaria Universitaria Parc Taulí, Sabadell, Spain; Microbiology Department, Centro Hospitalar de Lisboa Ocidental, Egas Moniz Hospital, Lisbon, Portugal

**Keywords:** Biomarkers, C-reactive protein, Procalcitonin, Mid-region fragment of pro-adrenomedullin, Ventilator-associated pneumonia, Clinical Pulmonary Infection Score, Diagnosis, Prediction

## Abstract

**Background:**

Prediction of diagnosis of ventilator-associated pneumonia (VAP) remains difficult. Our aim was to assess the value of biomarker kinetics in VAP prediction.

**Methods:**

We performed a prospective, multicenter, observational study to evaluate predictive accuracy of biomarker kinetics, namely C-reactive protein (CRP), procalcitonin (PCT), mid-region fragment of pro-adrenomedullin (MR-proADM), for VAP management in 211 patients receiving mechanical ventilation for >72 h. For the present analysis, we assessed all (*N* = 138) mechanically ventilated patients without an infection at admission. The kinetics of each variable, from day 1 to day 6 of mechanical ventilation, was assessed with each variable’s slopes (rate of biomarker change per day), highest level and maximum amplitude of variation (*Δ*^max^).

**Results:**

A total of 35 patients (25.4 %) developed a VAP and were compared with 70 non-infected controls (50.7 %). We excluded 33 patients (23.9 %) who developed a non-VAP nosocomial infection. Among the studied biomarkers, CRP and CRP ratio showed the best performance in VAP prediction. The slope of CRP change over time (adjusted odds ratio [aOR] 1.624, confidence interval [CI]_95%_ [1.206, 2.189], *p* = 0.001), the highest CRP ratio concentration (aOR 1.202, CI_95%_ [1.061, 1.363], *p* = 0.004) and *Δ*^max^ CRP (aOR 1.139, CI_95%_ [1.039, 1.248], *p* = 0.006), during the first 6 days of mechanical ventilation, were all significantly associated with VAP development. Both PCT and MR-proADM showed a poor predictive performance as well as temperature and white cell count.

**Conclusions:**

Our results suggest that in patients under mechanical ventilation, daily CRP monitoring was useful in VAP prediction.

*Trial registration* NCT02078999

**Electronic supplementary material:**

The online version of this article (doi:10.1186/s13613-016-0134-8) contains supplementary material, which is available to authorized users.

## Background

Ventilator-associated pneumonia (VAP) is usually caused by bacteria and is the most common serious intensive care unit (ICU)-acquired infection in patients undergoing invasive mechanical ventilation [1]. The widespread implementation of several preventive measures is, at least in part, associated with the observed decrease in VAP incidence [2].

One of the most challenging problems in VAP is its correct identification, resulting from the lack of a “gold standard” method of diagnosis [1]. The commonly used criteria are too sensitive but poorly specific [3]; as a result, up to 50 % of patients diagnosed with VAP do not have the condition and up to 30 % of cases of VAP are not correctly identified [4].

The accuracy of several biomarkers in the diagnosis and management of infection, namely VAP, have been evaluated repeatedly [5–9] with soluble triggering receptor expressed on myeloid cells (sTREM-1), C-reactive protein (CRP) and procalcitonin (PCT) being the most frequently studied.

The majority of the published studies assessed the usefulness of a single biomarker measurement in VAP diagnosis [3, 10]. Few have studied the value of serial measurements in the assessment of VAP, either before the diagnosis or after initiation of antibiotic therapy. In addition, these studies on biomarkers present discordant results [11–14], not achieving sufficient specificity or sensitivity to be routinely employed in clinical practice.

Our hypothesis was that the course of plasma concentrations of biomarkers after endotracheal intubation and invasive mechanical ventilation could be useful in VAP prediction. With that purpose, we assessed the predictive performance of kinetics of several biomarkers, namely CRP, PCT and MR-proADM, in all non-infected patients during the first 6 days of mechanical ventilation. Besides we also assessed the diagnostic performance of a single biomarker measurement at the day of VAP diagnosis.

## Methods

### Study design

The BioVAP study (biomarkers in the diagnosis and management of VAP) is a prospective, multicenter, observational study, designed to evaluate the additional information biomarkers can bring in the clinical decision-making process of VAP at the bedside (NCT02078999). The ICU recruitment was by direct invitation with no financial incentive. Local hospital ethics committees approved the study design, and written informed consent was obtained from all patients or their legally authorized surrogates in accordance with local requirements.

### Study subjects

**Fig. 1:**
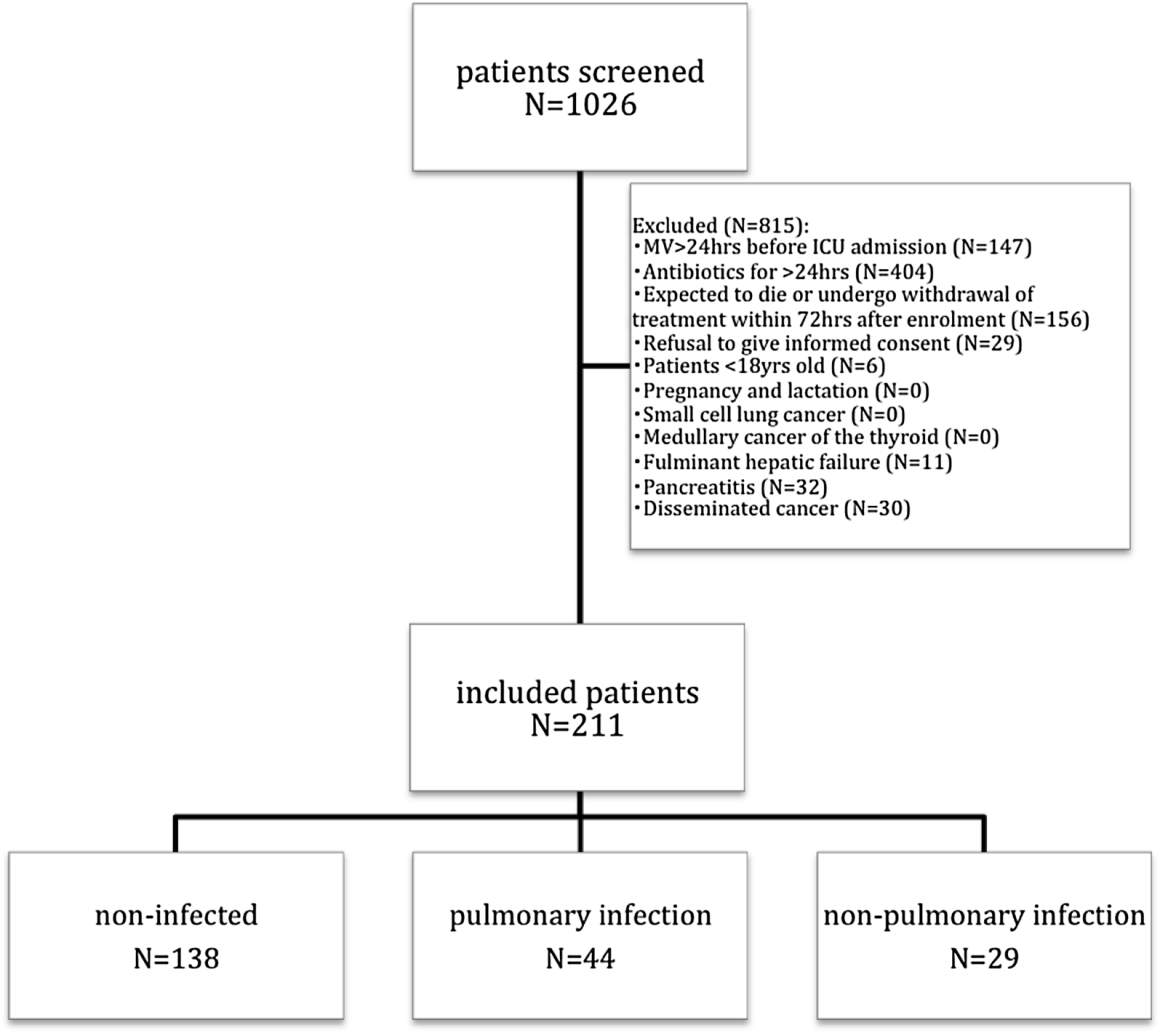
Flowchart of patients undergoing mechanical ventilation during the study period

During the study period (September 2008 till September 2010), all patients admitted to the participating ICU were screened for inclusion if they were mechanically ventilated for >72 h. A total of 211 included adult (>18 years) patients were divided into three groups: (1) non-infected, (2) pulmonary infection and (3) non-pulmonary infection (for details, see Fig. 1; Additional file 1). For each patient, only the first ICU admission and the first VAP episode were included in the study.

### Data collection and management

Data collection included demographic data and comorbid diseases (for data management, see Additional file 1). Clinical and laboratory data, namely the reason of mechanical ventilation at ICU admission, were recorded. The Simplified Acute Physiology Score (SAPS) II [15] was calculated from the worst values within the first 24 h after ICU admission. Microbiological and clinical infectious data were reported as well as the antibiotics prescribed, their changes and the duration of therapy. Organ dysfunctions were evaluated at ICU admission and during the duration of mechanical ventilation according to the Sequential Organ Failure Assessment (SOFA) score [16].

Day 1 (D1) was considered the day of initiation of invasive mechanical ventilation. Patients were monitored till D21, the day of successful weaning and extubation, the day of a non-VAP infection or the day of clinical diagnosis of VAP whatever arrived first (for definitions see Additional file 1).

The following clinical variables were collected daily: mechanical ventilation parameters at 08:00, American College of Chest Physicians/Society of Critical Care Medicine (ACCP/SCCM) consensus conference on sepsis criteria, simplified Clinical Pulmonary Infection Score (CPIS) [17, 18], SOFA score, daily registry of the renal support therapy, surgery (type and reason), steroids (drug, dose and reason), any ICU-acquired infection other than VAP, antibiotic therapy if applied.

Blood samples were obtained from an arterial line at ICU admission and subsequently daily every morning for the routine assessment of CRP, PCT, MR-proADM and arterial blood gases. In all patients, a quantitative tracheal aspirate (QTA) was performed at ICU admission and subsequently twice a week (Mondays–Thursdays or Tuesdays–Fridays).

Patients were followed up till death or ICU discharge as well as hospital discharge. At 90th day, a telephonic interview was performed for outcome assessment.

For the present analysis, we compared biomarker kinetics of VAP patients and non-infected controls during the first 6 days of mechanical ventilation as well as at the day of VAP diagnosis. All VAP have microbiological documentation (for definitions, see Additional file 1).

### Statistical analysis

Continuous variables were expressed as mean and standard deviation (SD) or median and interquartile range (IQR) if the distribution was clearly asymmetric. Comparisons between groups were performed with two-tailed unpaired Student’s *t* test or Mann–Whitney *U* tests for continuous variables according to data distribution. Fisher’s exact test and Chi-square test were used to carry out comparisons between categorical variables as appropriate.

In addition to CRP evaluation, we also assessed the relative changes in CRP concentration and the CRP ratio. The relative changes were calculated in relation to D1 CRP concentration.

Time-dependent analysis of different variables from D1 to D6 of mechanical ventilation was performed with general linear models univariate repeated measures analysis using a split-plot design approach.

For the statistical analysis of the patient’s infectious status, VAP versus non-infected controls, as function of a longitudinal covariate, obtained from the six measurements of the variables of interest between D1 and D6 (CRP, PCT, MR-proADM, white cell count (WCC), temperature and CPIS), we used a two-step approach as previously described elsewhere [19] (for additional details, see Additional file 1), in order to evaluate the slope of each variable over time (see Additional file 2: Figure S1).

Receiver operating characteristics curves (ROC) were plotted for the day of VAP diagnosis of the studied variables. The accuracy of these variables was assessed calculating its area under the curve (AUC), assessment of the best cutoff value, sensitivity and specificity calculation as well as the likelihood ratios.

Data were analyzed using PASW version 20.0 for MAC (SPSS, Chicago, IL, USA) and R (R Development Core Team: A Language and Environment for Statistical Computing. Vienna, Austria: 2005). Adjusted odds ratios (OR) with 95 % confidence interval (CI) were computed. All statistics were two-tailed, and significance level was set at 0.05.

## Results

During the study period, a total of 211 patients were included in the BioVAP study (Fig. 1). For the present analysis, we assessed all non-infected mechanically ventilated patients (*N* = 138). A total of 35 patients (25.4 %) developed VAP, 70 (50.7 %) had no infection and did not receive antibiotics (controls), and 33 (23.9 %) developed another non-VAP nosocomial infection. The last group was excluded.

The baseline patients’ characteristics are presented in Table 1. At the day of initiation of mechanical ventilation, patients that develop a VAP and non-infected controls presented similar characteristics, with the two exceptions; at admission, in VAP group, CRP was significantly lower and SOFA score was significantly higher when compared with controls. From the 35 VAP episodes (35/211—25.4 %), with 41 bacterial isolates (see Additional file 1), 18 were early VAP and 77.1 % were diagnosed during the first week of mechanical ventilation. The duration of mechanical ventilation till the diagnosis of VAP was (median) 5.0 days (IQR 4.0).

**Table 1:** Baseline characteristics of all patients mechanically ventilated for non-infectious reasons

	Total (*N* = 138)	VAP (*N* = 35)	No infection (*N* = 70)	*p*
Male, *N* (%)	93 (67.4 %)	26 (74.3 %)	41 (58.6 %)	0.116
Age (years)	59.8 ± 18.4	57.9 ± 16.2	60.6 ± 20.5	0.501
SAPS II	49.1 ± 18.4	52.6 ± 18.3	49.8 ± 19.0	0.479
SOFA	7.2 ± 3.0	8.1 ± 2.9	6.8 ± 2.9	0.045
CPIS	2.6 ± 1.9	2.7 ± 2.0	2.7 ± 1.9	0.971
Cause of admission, *N* (%)				0.581
Medical	96 (69.6 %)	25 (71.4 %)	50 (71.4 %)	
Trauma	2 (1.4 %)	8 (22.9 %)	11 (15.7 %)	
Elective surgery	27 (19.6 %)	0	1 (1.4 %)	
Emergency surgery	13 (9.4 %)	2 (5.7 %)	8 (11.4 %)	
Comorbidities, *N* (%)				
COPD	19 (13.8 %)	7 (20.0 %)	6 (8.6 %)	0.119
Steroids	1 (0.7 %)		1 (1.4 %)	
Diabetes	19 (13.8 %)	3 (8.6 %)	12 (17.1 %)	0.375
Immunosuppression	3 (2.2 %)		1 (1.4 %)	
CHF	23 (16.7 %)	3 (8.6 %)	14 (20.0 %)	0.167
CLD	1 (0.7 %)	1 (2.9 %)		
CRF	9 (6.5 %)	3 (8.6 %)	6 (8.6 %)	1.0
HIV	3 (2.2 %)	1 (2.9 %)	2 (2.9 %)	1.0
Admission diagnosis, *N* (%)				0.501
CVA	16	6	10	
AECB	6	2	4	
Decompensated CHF	17	5	12	
TBI	19	10	9	
Others	28	14	14	
Reason of MV, *N* (%)				0.1
Respiratory failure	40 (29.0 %)	8 (22.9 %)	23 (32.9 %)	
Shock	17 (12.3 %)	8 (22.9 %)	5 (7.1 %)	
Coma	76 (55.1 %)	17 (48.6 %)	40 (51.7 %)	
Other	5 (3.6 %)	2 (5.7 %)	2 (2.9 %)	
Tidal volume (mL)	458 [146]	488 [97]	442 [160]	0.21
Plateau pressure (cmH_2_O)	19 [7]	21 [9]	19 [6]	0.213
PEEP	5 [2]	5 [3]	5 [2]	0.686
PaO_2_/FiO_2_	245 [172]	245 [122]	224 [213]	0.828
CPR (mg/dL)	6.00 [8.62]	4.33 [6.20]	8.40 [9.39]	0.003
PCT (μg/L)	0.40 [1.76]	0.94 [2.37]	0.34 [1.48]	0.167
MR-proADM (nmol/L)	1.85 [2.64]	1.70 [2.87]	1.91 [2.82]	0.470
WCC (×10^3^/mm^3^)	12.46 ± 4.55	12.58 ± 4.92	11.85 ± 4.54	0.456
Temperature (°C)	36.7 ± 1.3	36.9 ± 1.3	36.4 ± 1.3	0.126
Nosocomial infection^a^	68 (49.3 %)			
VAP	35 (25.4 %)			
VAT	14 (10.1 %)			
CVC bacteremia	2 (1.4 %)			
UTI	6 (4.3 %)			
Surgical infection	5 (3.6 %)			
Other	6 (4.3 %)			
Duration of MV (days)	7.5 [9.8]	14.0 [8.0]	5.0 [5.5]	<0.001
LOS ICU (days)	12.0 [12.0]	18.0 [12.0]	10.0 [8.5]	<0.001
LOS hospital (days)	25.0 [30.3]	27.0 [31.5]	24.0 [30.5]	0.55
Mortality D28, *N* (%)	16 (18.6)	15 (40.5)	1 (2)	<0.001
Mortality D90, *N* (%)	20 (23.3)	15 (40.5)	5 (10.2)	0.004

*AECB* acute exacerbation of chronic bronchitis, *CHF* chronic heart failure, *CVA* cerebrovascular accident, *CLD* chronic liver disease, *COPD* chronic obstructive pulmonary disease, *simplified CPIS* Clinical Pulmonary Infection Score, *CRF* chronic renal failure, *CRP* C-reactive protein, *CVC* central venous catheter, *HIV* human immunodeficiency virus, *ICU* intensive care unit, *LOS* length of stay, *MV* mechanical ventilation, *MR*-*proADM* mid-region fragment of pro-adrenomedullin, *PaO*_*2*_ */FiO*_*2*_ ratio of partial pressure of arterial O_2_ to the fraction of inspired O_2_, *PCT* procalcitonin, *PEEP* positive end-expiratory pressure, *SAPS* Simplified Acute Physiology Score, *SOFA* Sequential Organ Failure Assessment, *TBI* traumatic brain injury, *UTI* urinary tract infection, *VAP* ventilator-associated pneumonia, *VAT* ventilator-associated tracheobronchitis, *WCC* white cell count

### Kinetics of biomarkers and inflammatory variables

**Fig. 2:**
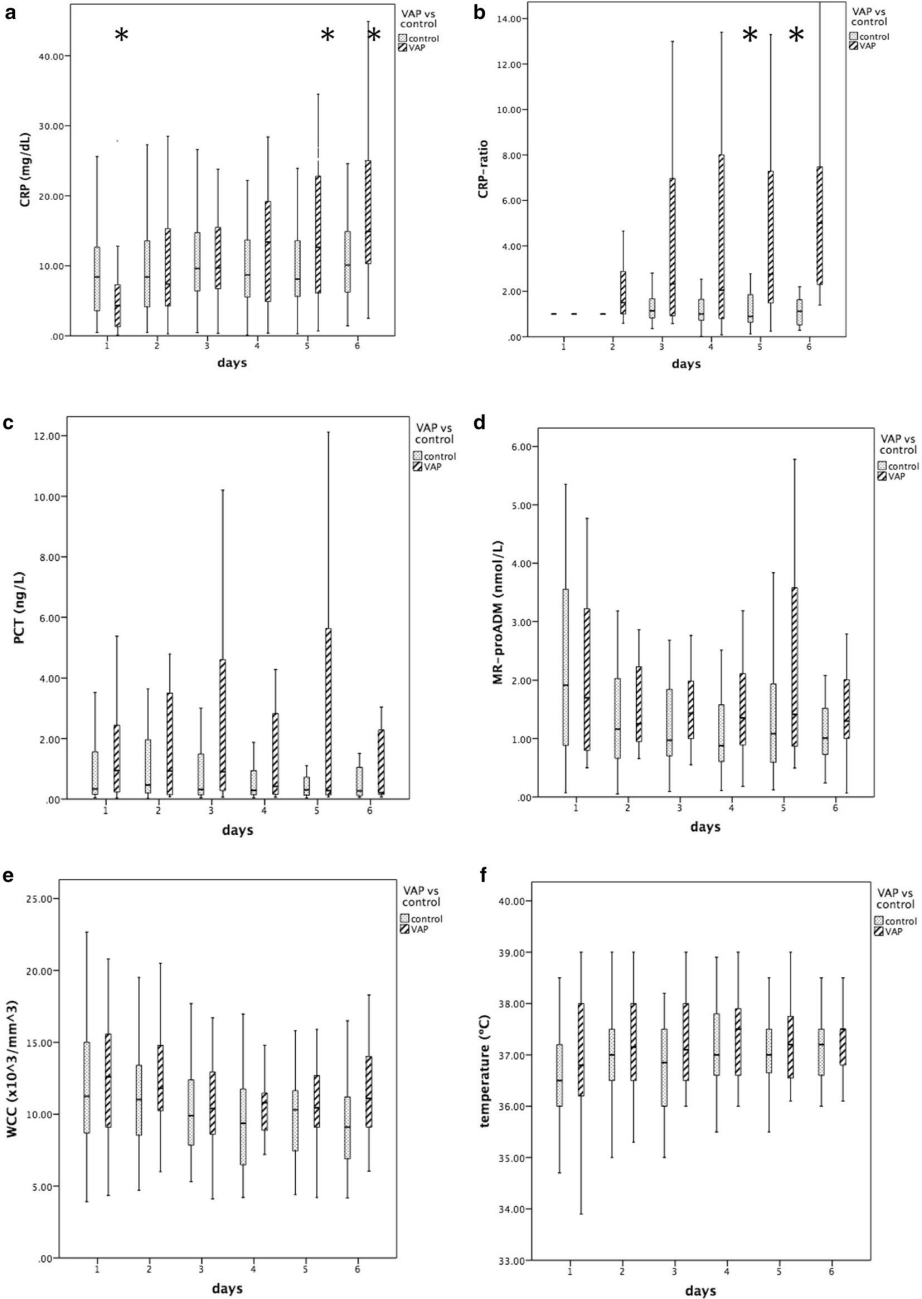
Time course of biomarkers (CRP, PCT and MR-proADM), temperature and WCC from day 1 to day 6 of mechanical ventilation in ventilator-associated pneumonia (VAP) patients and non-infected controls (**a** CRP, **b** CRP ratio, **c** PCT, **d** MR-proADM, **e** WCC, **f** temperature). Time-dependent analysis of CRP, CRP ratio and CPIS was significantly different between VAP patients and controls (*p* < 0.001, *p* < 0.001 and *p* = 0.019, respectively). Some variables, namely CRP and CRP ratio, became significantly higher by day 5 in patients that will develop a VAP in comparison with controls (*p < 0.05). *CRP* C-reactive protein, *MR*-*proADM* mid-region fragment of pro-adrenomedullin, *PCT* procalcitonin, *VAP* ventilator-associated pneumonia, *WCC* white cell count

Figure 2 presents the variables’ values during the study period from D1 to D6. The time-dependent analysis of CRP and CRP ratio was significantly different between non-infected controls and patients that went on to develop a VAP (*p* < 0.001 and *p* < 0.001, respectively). In VAP patients, we found no differences in CRP kinetics between early and late VAP (*p* = 0.304). When we compared CRP and CRP ratio at the different time points, their values were significantly higher from D5 of mechanical ventilation onwards in VAP patients. The time-dependent analysis of PCT (log transform), MR-proADM, WCC and temperature values was not significantly different between groups (*p* = 0.685, *p* = 0.753, *p* = 0.681 and *p* = 0.835, respectively).

To study the value in VAP prediction of the kinetics of each variable, we evaluated the absolute changes from D1 to D6 of mechanical ventilation assessed with the previously calculated slopes, as well the highest value and the *Δ*^max^.

**Fig. 3:**
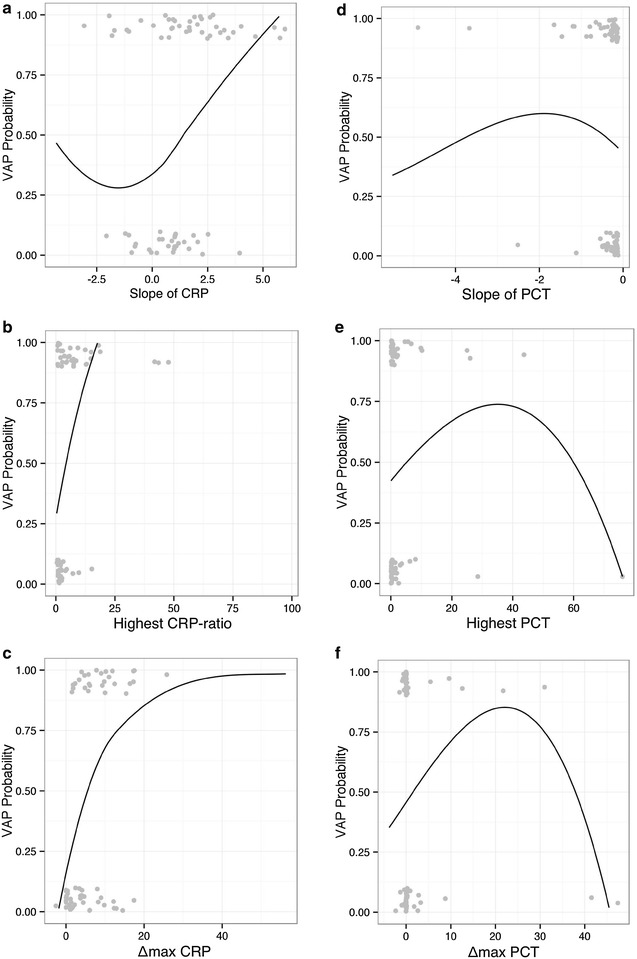
Curve of disease risk probability of ventilator-associated pneumonia (VAP), for the possible range of kinetics of CRP concentration changes over time, assessed by the slope, highest value and *Δ* ^max^ from day 1 to day 6 of mechanical ventilation (**a**–**c** CRP, **d**–**f** PCT, respectively). Ideally, the *line* should show a linear relationship between the marker and the probability of VAP. For PCT, the same calibration plots are presented (slope, highest and *Δ* ^max^). *CRP* C-reactive protein, *VAP* ventilator-associated pneumonia

The slope describes the rate of change per day of a particular variable in each patient from the beginning of mechanical ventilation, that is D1, till D6. Additional file 2: Figure S1 presents some examples of predictions of individual CRP slopes that describe the CRP rate of change per day in an individual patient. Among all the studied slopes (Additional file 1), only CRP and CRP ratio were significantly different between groups (*p* = 0.001, *p* < 0.001, respectively). The slopes of CRP and CRP ratio showed a reasonable diagnostic performance with a ROC–AUC >0.7. Besides, CRP and CRP ratio were significantly associated with VAP prediction (Table 2). After adjustment for confounders, the slope of CRP was significantly associated with VAP development (aOR 1.624, CI_95%_ [1.206, 2.189], *p* = 0.001). The ability of the model to predict VAP assessed by the area under the ROC curve was 0.71 (CI_95%_ [0.60; 0.82]). As an example, a patient with an average increase in CRP concentration of 1 mg/dL/day from D1 till D6 of mechanical ventilation has 62 % greater chance of having VAP when compared with a patient with no CRP increase. The same is shown in Fig. 3, with the CRP-slope calibration plot showing that the higher the slope, the higher the VAP probability.

**Table 2:** Evaluation of studied variables in ventilator-associated pneumonia prediction

	OR	95 % CI	*p*	aOR	95 % CI	*p*
Slope						
CRP (mg/dL)	1.641	1.229–2.192	<0.001	1.624	1.206–2.189	0.001
CRP ratio	1.516	1.021–2.250	0.039	1.480	1.060–2.067	0.021
PCT (μg/L)	0.803	0.544–1.183	0.267	0.844	0.559–1.274	0.419
ADM (nmol/L)	0.740	0.147–3.742	0.716	0.730	0.137–3.902	0.713
WCC (×10^3^/mm^3^)	1.182	0.807–1.729	0.391	1.225	0.809–1.855	0.338
Temperature (°C)	0.288	0.033–2.540	0.262	0.270	0.028–2.590	0.256
Highest						
CRP (mg/dL)	1.044	1.000–1.090	0.052	1.037	0.992–1.085	0.11
CRP ratio	1.201	1.065–1.355	0.003	1.202	1.061–1.363	0.004
PCT (μg/L)	1.032	0.987–1.079	0.168	1.020	0.974–1.068	0.392
ADM (nmol/L)	1.335	1.022–1.744	0.034	1.369	1.035–1.809	0.028
WCC (×10^3^/mm^3^)	1.032	0.987–1.079	0.168	1.020	0.974–1.068	0.392
Temperature (°C)	2.043	1.170–3.536	0.012	2.053	1.126–3.744	0.019
*Δ* ^max^						
CRP (mg/dL)	1.151	1.057–1.252	0.001	1.139	1.039–1.248	0.006
CRP ratio	1.213	1.030–1.428	0.021	1.186	1.018–1.381	0.029
PCT (μg/L)	1.036	0.984–1.089	0.178	1.023	0.971–1.078	0.399
ADM (nmol/L)	1.395	0.964–2.020	0.078	1.372	0.943–1.996	0.099
WCC (×10^3^/mm^3^)	1.044	0.963–1.131	0.294	1.046	0.959–1.140	0.312
Temperature (°C)	1.020	0.665–1.565	0.928	0.933	0.583–1.494	0.772

Variables included in the adjusted model: age, sex, SAPS II, cause of admission

*Simplified CPIS* Clinical Pulmonary Infection Score, *CRP* C-reactive protein, *MR*-*proADM* mid-region fragment of pro-adrenomedullin, *OR* odds ratio, *PCT* procalcitonin, *ROC* receiver operating characteristics, *VAP* ventilator-associated pneumonia, *WCC* white cell count

We evaluated the highest value reached by a variable from the beginning of mechanical ventilation, that is D1, till D6 in VAP patients and non-infected controls. Of the studied variables (Additional file 1), only highest CRP ratio, MR-proADM and temperature were significantly different between groups (*p* < 0.001, *p* = 0.014, *p* = 0.027, respectively). However, only the highest value of CRP ratio showed a reasonable diagnostic performance with a ROC–AUC above 0.7. Moreover, the highest value of CRP ratio and MR-proADM were significantly associated with VAP prediction (Table 2). After adjustment for confounders, highest CRP ratio was significantly associated with VAP development (aOR 1.202, CI_95%_ [1.061, 1.363], *p* = 0.004). The ability of the model to predict VAP assessed by the area under the ROC curve was 0.75 (CI_95%_ [0.64; 0.87]) for the highest CRP ratio. As an example, for each 10 % increase in the highest CRP ratio concentration from D1 to D6 was associated with a 20 % greater chance of having VAP when compared with a patient with no CRP ratio change. The same is shown in Fig. 3, with the calibration plot showing that the higher the highest CRP ratio, the higher the VAP probability.

The maximum delta (*Δ*^max^) evaluates the difference between the lowest and the highest value of each variable from the beginning of mechanical ventilation, that is D1, till D6 in VAP patients and non-infected controls. Of the studied variables (Additional file 1), only CRP, CRP ratio and MR-proADM were significantly different between groups (*p* < 0.001, *p* < 0.001, *p* = 0.01, respectively). The *Δ*^max^ of CRP showed a good diagnostic performance with a ROC–AUC >0.75 but was outperformed by *Δ*^max^ of CRP ratio with an ROC–AUC of 0.82. Besides, *Δ*^max^ of CRP and CRP ratio was significantly associated with VAP prediction (Table 2). After adjustment for confounders, *Δ*^max^ of CRP was significantly associated with VAP development (aOR 1.139, CI_95%_ [1.039, 1.248], *p* = 0.006, respectively). The ability of the adjusted model to predict VAP assessed by the area under the ROC curve was 0.82 (CI_95%_ [0.73; 0.91]) for *Δ*^max^ CRP. As an example, for each 1 mg/dL increment in *Δ*^max^ CRP concentration from D1 to D6 of mechanical ventilation was associated with a 14 % greater chance of having VAP when compared with a patient with no CRP concentration change.

Figure 3 also shows the calibration plots of PCT (slope, highest and *Δ*^max^). The inverted U-shape of the three curves clearly shows that the kinetics of PCT assessed by the slope, the highest value as well as the *Δ*^max^ was not useful in VAP prediction that is far from the ideal linear correlation that is indicative of a good diagnostic marker.

## Discussion

The present analysis of the BioVAP study showed that, among the studied biomarkers, only the kinetics of serial CRP measurements during the first 6 days of mechanical ventilation was useful in VAP prediction. We showed that the rate of change per day of CRP and CRP ratio, but also the highest value of CRP ratio and the maximum change in CRP and CRP ratio during the study period were all associated with VAP prediction. In addition, at the day of VAP diagnosis we showed that a single measurement of CRP was useful in particular to exclude VAP diagnosis, whereas CPIS was better to include VAP.

In our analysis, VAP and controls presented similar baseline characteristics, with the exception of CRP, which was higher in the control group, and SOFA score, which was higher in patients that developed VAP. Our study was not designed to assess mortality or prognosis of mechanically ventilated patients but to evaluate the predictive performance for VAP of the kinetics of different biomarkers, that is to say, to identify patients with a high probability VAP, before its clinical diagnosis. To do so, we assessed the kinetics of several biomarkers during the first 6 days of mechanical ventilation to evaluate their performance in VAP prediction, not as risk factors [20].

In VAP, some studies have previously looked at PCT and/or CRP concentration changes before diagnosis. Luyt et al. [13] found that PCT, either absolute values or concentration changes in the 5 days before diagnosis, had a poor diagnostic performance for late VAP. Charles et al. [21] showed that, within the period spanning 3 days before the day of diagnosis, PCT changed only in the last 24 h but with a very good diagnostic performance. Finally, in the diagnosis of aspiration syndromes, PCT presented a poor diagnostic performance in three studies [22–24], whereas CRP showed to be clinically helpful in the one study in which it was evaluated [22]. None of these studies performed an analysis of biomarker kinetics.

In our study, it was also clear that, from D1 to D6 of mechanical ventilation, both CRP and CRP ratio increase steadily in VAP group and became significantly higher from D5 onwards. Of the studied biomarkers, the slope of CRP was the individual variable most useful in VAP prediction. In addition, the calibration plot nicely separates those patients with low slopes and low probability of VAP from those with higher slopes and higher probability. This is particularly relevant, since monitoring the course of a single biomarker is much easier to interpret than the calculation of a score.

Besides, we also evaluated a single biomarker measurement on the day of VAP diagnosis. In doing so, we found that CRP was the best biomarker. With a similar methodology, we have previously showed that CRP presented a good performance in VAP diagnosis, and interestingly found an almost equal cutoff of 9.6 mg/dL [25].

As far as we are aware, the present study is the first using strict inclusion criteria comparing the diagnostic performance of CRP and PCT in VAP prediction. Previous studies comparing the diagnostic performance for infection of a single value of CRP and PCT in different clinical scenarios showed that, at the day of diagnosis, the AUC of CRP was always higher than of PCT [22, 26–31]. Similarly, in our study in VAP patients, CRP performed better than PCT, presenting a good negative likelihood ratio, meaning that CRP is a good biomarker to exclude that diagnosis in the presence of dubious clinical manifestations. On the opposite, the poor diagnostic performance of PCT has been attributed to the relatively low virulence of the usual microorganisms found in VAP [8, 32] as well as being considered a compartmentalized infection [32, 33].

The MR-proADM is a novel biomarker and has not yet been well evaluated in the diagnosis of infection [34], and never before in VAP. In our study, MR-proADM showed a poor diagnostic performance for VAP.

At the day of VAP diagnosis, CPIS showed to be superior to any of the studied biomarkers. It had an excellent diagnostic performance, namely a high positive likelihood ratio. Regarding CPIS, there is still controversy concerning its widespread clinical use as a surrogate of VAP diagnosis since it lacks well-done studies of validation as well as marked inter-observer variability [35]. Besides, the routine use of CPIS is time-consuming and not possible to be mentally calculated; however, in the upside, it is an inexpensive tool.

Our study has several strengths. First, this is a multicenter prospective observational study that limits the potential bias of reflecting only the practice from one center, as well as from retrospective data collection. Second, we followed a group of patients ventilated for non-infectious reasons during the first 6 days of mechanical ventilation. This way we could assess the kinetics of biomarkers in VAP prediction.

Besides, we recognize that the present study has limitations. First, the nonrandomized and observational nature of the study design bears the potential of unmeasured confounders that may have caused differences in therapeutic and supportive approach. Second, despite the use of quantitative cultures in VAP diagnosis, it is important to consider the potential false-positive and false-negative rates of quantitative cultures that could have influenced the results. Third, our findings can only be applied to non-infected patients admitted in the ICU for invasive MV and on MV for >72 h. Fourth, since measurement CRP was available daily in all centers and PCT in three centers, this could have introduced potential unmeasured bias in patient classification. Finally, it is possible that other types of patients as well as infections, not VAP, may have different biomarker time courses. Thus, the presence of non-pulmonary infections besides VAP may influence the diagnostic accuracy we report in this manuscript. Besides, our results are not applicable to patients that have a previous infection since we compared cases initially non-infected to non-infected controls.

## Conclusions

In summary, we found that the combination of two cheap and widely available tools, CRP and CPIS, could be very helpful in the approach of patients undergoing mechanical ventilation with risk of VAP but always in combination with the available clinical data. On the one hand, we showed that the kinetics of CRP in the days before VAP diagnosis, namely the slope of CRP, could be useful in VAP prediction. A patient with an average increase in CRP of 1 mg/dL/day has 62 % greater chance of having a VAP when compared with a patient with no CRP increase during the first 6 days of mechanical ventilation. In addition, on the day of VAP, we could use CRP and CPIS, since CRP could be useful to exclude VAP diagnosis and CPIS to admit the diagnosis. As a result, this innovative approach needs to be further validated in different settings and with a larger sample size.

## Appendix

*Study coordinators* Antonio Artigas Raventós (Area de Críticos, Corporació Sanitaria Universitaria Parc Taulí, Hospital de Sabadell, CIBERES, Sabadell, Spain), Pedro Póvoa (Unidade de Cuidados Intensivos Polivalente, Hospital São Francisco Xavier, Centro Hospitalar de Lisboa Ocidental, NOVA Medical School, New University of Lisbon, Lisbon, Portugal).

*Participating intensive care units* Corporación Sanitaria Universitaria Parc Taulí, Hospital de Sabadell, Sabadell, Spain (Ignacio Martin-Loeches, Eugenio Berlanga, Mateu Espasa, Gisela Gili, Gemma Goma), Unidade de Cuidados Intensivos Polivalente, Hospital São Francisco Xavier, Centro Hospitalar de Lisboa Ocidental, Lisbon, Portugal (Joana Silvestre, Elsa Gonçalves), Hospital Universitario y Politécnico La Fe, Valencia, Spain (Paula Ramirez), Hospital Clinic Provincial, Barcelona, Spain (Antoni Torres, Mariano Esperati).

## Additional files

10.1186/s13613-016-0134-8 Electronic supplemental material
10.1186/s13613-016-0134-8 Observed values of CRP from day 1 to day 6 of mechanical ventilation and the predicted slopes by the model for a randomly selected group of patients

## Abbreviations

AUC: area under the curve
CI: confidence interval
CPIS: Clinical Pulmonary Infection Score
CRP: C-reactive protein
ICU: intensive care unit
OR: odds ratio
MR-proADM: mid-region fragment of pro-adrenomedullin
MV: mechanical ventilation
PCT: procalcitonin
QTA: quantitative tracheal aspirate
ROC: receiver operating characteristic
SAPS: Simplified Acute Physiology Score
SOFA: Sequential Organ Failure Assessment
VAP: ventilator-associated pneumonia
WCC: white cell count
