# Supplementary material for: Biomarker kinetics in the prediction of VAP diagnosis: results from the BioVAP study
Source: Ann Intensive Care. 2016 Apr 14;6:32. doi: 10.1186/s13613-016-0134-8 (PMC4830786; doi:10.1186/s13613-016-0134-8)
Supplement: Supplementary file 1 — 10.1186/s13613-016-0134-8 Electronic supplemental material [file 13613_2016_134_MOESM1_ESM.doc]

**Title: Biomarker kinetics in the prediction of VAP diagnosis; results from the BioVAP study**

Electronic Supplemental Material

**Authors**: Pedro Póvoa^1,2^, Ignacio Martin-Loeches^3,4^, Paula Ramirez^4,5^, Lieuwe D. Bos^6^, Mariano Esperatti^4,7^, Joana Silvestre^1,2^, Gisela Gili^3,4^, Gema Goma^3,4^, Eugenio Berlanga^8^, Mateu Espasa^8^, Elsa Gonçalves^2,9^, Antoni Torres^4,7^, Antonio Artigas^3,4^

**Affiliations**:

^1^ Polyvalent Intensive Care Unit, São Francisco Xavier Hospital, Centro Hospitalar de Lisboa Ocidental, Lisbon, Portugal

^2^ NOVA Medical School, CEDOC, New University of Lisbon, Lisbon, Portugal

^3^ Critical Care Center, Sabadell Hospital, Corporación Sanitaria Universitaria Parc Taulí, Universitat Autonoma de Barcelona, Sabadell, Spain

^4^ CIBER de Enfermedades Respiratorias (CIBERES), Madrid, Spain

^5^ Intensive Care Unit, University Hospital La Fe, Valencia, Spain

^6^ Department of Intensive Care, Academic Medical Center, University of Amsterdam, Amsterdam, The Neherlands

^7^ Respiratory Disease Department, Hospital Clínic i Provincial de Barcelona, IDIBAPS, Barcelona, Spain

^8^ Laboratory Department, UDIAT, Corporación Sanitaria Universitaria Parc Taulí, Sabadell, Spain

^9^ Microbiology Department, Egas Moniz Hospital, Centro Hospitalar de Lisboa Ocidental, Lisbon, Portugal

**Material and Methods**

*Study subjects*

A total of 211 included adult (>18 yrs) patients were divided into 3 groups: 1) non-infected – admitted in the ICU for mechanical ventilation for a non-infectious cause of respiratory failure, without evidence of pneumonia in the chest x-ray, that were not receiving antibiotics for at least 5 days before ICU admission, with an expected length of mechanical ventilation >72hrs and in whom antibiotics were not prescribed on admission by the attending physician (the use of antibiotics as prophylaxis was not an exclusion criteria); 2) pulmonary infection – admitted in the ICU for mechanical ventilation with a clinical diagnosis of community-acquired or hospital-acquired pneumonia, that were not receiving antibiotics for >24hrs before ICU admission, with an expected length of mechanical ventilation >72hrs; 3) non-pulmonary infection – the same as 2) but with a clinical diagnosis of community or hospital-acquired non-pulmonary infection. Non-inclusion criteria were mechanical ventilation >24hrs before ICU admission, antibiotics for >24hrs for a pulmonary or extra-pulmonary infection, expected to die or undergo withdrawal of treatment within 72hrs after enrolment, refusal to give informed consent, patients <18yrs old, pregnancy and lactation, small cell lung cancer, medullary cancer of the thyroid, fulminant hepatic failure, pancreatitis, disseminated cancer. The inclusion and exclusion criteria of BIOVAP study followed the recommendations of the European Society of Intensive Care Medicine [1] as well as several published papers [2-4] for the design of a study to assess the diagnostic performance of a biomarker of infection.

*Data management*

Data were collected prospectively either using pre-printed case report forms or a specific database software. All data were collected in a central database located at the Critical Care Centre of Sabadell Hospital, Corporación Sanitaria Universitaria Parc Taulí, Sabadell, Spain. Detailed instructions concerning the aims of the study and data collection were given to all participating centers, before starting data collection and throughout the study period. A medical doctor was individually designated as responsible for data collection and quality control in each intensive care unit (ICU). Data was also screened in detail by two different persons (IML and GG) for missing information, implausible and outlying values. The participating centers had a constant feedback of the patients included, as well as, any problems related with the data since the program was designed to identify and reject inconsistencies. The study coordinators were easily accessible to all participating investigators by phone or email to answer all queries during the study.

*Definitions*

Infection was defined as a pathologic process caused by the invasion of normally sterile tissue or fluid or body cavity by a pathogenic or potentially pathogenic microorganism [5] and/or clinically suspected infection, plus the prescription of antimicrobial therapy. Community-acquired infection, either of pulmonary or extra-pulmonary origin, was defined as the onset of infection prior to hospital admission or not present at admission but becomes evident in the first 48hrs [6]. All infections diagnosed after 48hrs of hospital stay were classified as hospital-acquired.

Presence of sepsis was defined according to the American College of Chest Physicians/Society of Critical Care Medicine Consensus Conference criteria [7]. Severe sepsis and septic shock were defined as published elsewhere [5, 7]. Emergency surgery was defined as a non-scheduled surgery within 24hrs prior to ICU admission.

Patients admitted in the ICU for mechanical ventilation with a clinical diagnosis of community-acquired pneumonia (CAP) or hospital-acquired pneumonia (HAP) should present the following criteria: a new and persistent radiographic pulmonary infiltrate plus at least 2 of the following criteria: a) temperature >38°C or <36°C; b) white cell count (WCC) >10 or <4 x 10^3^/mm^3^; c) purulent tracheal aspirate [8, 9]. In addition, we perform a quantitative tracheal aspirate (QTA), a bronchoscopic or non-bronchospcopic broncho-alveolar lavage (BAL) and at least two separate blood cultures. In patients with CAP suspicion pneumococcal and *Legionella pneumophila* serogroup 1 antigen urine tests were performed. Empiric antibiotic therapy should be started according to the American Thoracic Society or to the Hospital Clínic of Barcelona guidelines of ventilator-associated pneumonia (VAP) treatment [10, 11].

In patients admitted in the ICU for mechanical ventilation with a clinical diagnosis of community or hospital-acquired infection, without evidence of pneumonia in the chest x-ray, empiric antibiotic therapy should be started according the presumed infection site as well as the most probable causative microorganisms.

Patients were classified according to their primary admission diagnosis into four categories: medical, trauma, elective surgery and emergency surgery. The reason for mechanical ventilation was classified as respiratory failure, coma, shock and other.

Clinical diagnosis of VAP was defined by the same criteria for CAP or HAP in patients under mechanical ventilation for at least 48hrs. The chest x-rays were reviewed either by the attending physicians or a radiologist. In case of disagreement, a third physician was asked to interpret the chest X-ray. Patients being diagnosed as having VAP need to have a new infiltrate in the day of the diagnosis and the following days. In addition, it was mandatory to perform a QTA, a bronchoscopic or non-bronchospcopic BAL and at least two separate blood cultures [12, 13]. The thresholds used for diagnosis of pneumonia were ≥10^5^ colony-forming units (cfu)/ml on a QTA and ≥10^4^ cfu/ml of on a BAL. Empiric antibiotic therapy should be started according to the above-mentioned guidelines. It was acceptable to adjust empiric antibiotic therapy according to any QTA previous findings, as well as, the prevalent ICU microflora in each participating centre.

*Samples collection*

Plasma samples were collected every day and deep-frozen (4 ml of blood collected in vacuum tubes, centrifuged 3,000 rpm for 10 min, plasma samples of 1 to 1.5 ml in two tubes for deep freeze at -80°C for posterior analysis).

In addition, at the days of QTA, as well as in the case of BAL sample, an aliquot was separated and deep-frozen (-80°C) for future measurements.

*Biomarkers Assays*

C-reactive protein was measured daily in all centres and procalcitonin (PCT) was also available in three. Mid-region fragment of pro-adrenomedullin (MR-proADM) samples were deep-frozen until processing and also with PCT of the remaining center. Circulating plasma CRP levels were measured with a commercially available kit using a particle enhanced turbidimetric immunoassay technique with an assay sensitivity of 0.05 mg/dL. Circulating plasma PCT and MR-proADM levels were measured with a TRACE™ technology assay (Time-Resolved Amplified Cryptate Emission), Brahms AG, Hennigsdorf, Germany.

*Assessment of sample size*

Under the assumptions of an expected sensitivity and specificity of 90% for the PCT test diagnosis, a VAP incidence of 15-18%, setting precision at 7% and confidence level at 95%, we would need a sample size of 395 patients, in order to reach more than 50 documented VAP, that was considered to be a good sample size. The study was scheduled to last one year and to include 6 centers; however due to logistic problems, 2 centers were excluded. Besides, during the first year, the participating centers presented an expectedly low VAP rate (<10%). As a result, the Steering Committee decided to extend the study for one year more. The inclusion was stopped in September 2010 with the inclusion of 211 patients.

*Statistical Analysis*

For the statistical analysis of the patient’s infectious status, VAP versus controls, as a function of a longitudinal covariate (measurements of a variable from day 1 to day 6 of mechanical ventilation) we used a two-steps approach as previously described [14]. First, we modeled each variable measurements as a function of time (6 days of measurement). We used a linear mixed model which allowed us to predict an intercept and a slope for each patient. This step is conceptually similar to fitting a linear regression, Mean_ “variable”=α+β*day, for each patient and obtaining an individual intercept (α) and slope (β) per patient [15]. Figure 1 ESM shows the observed values of CRP and predicted slopes by the model for a randomly selected group of patients. Then, we used the individual slope of the variable as covariates in a logistic regression for VAP diagnosis. This model allowed us to compute the odds ratios associated with each slope of a variable that subsquently were adjusted for patient’s age, sex, SAPS II and admission diagnosis. For all logistic models, we checked the Hosmer and Lemeshow goodness-of-fit and computed the receiver operating characteristic curve to evaluate model discrimination.

**Results**

*Microbiology of VAP*

We diagnosed a total of 35 VAP episodes (6 polymicrobial) with 41 isolates (51.5% Gram positive, p=0.276) (table 1 ESM). Early VAP were more frequently caused by Gram positive bacteria whereas late episodes were more frequently caused by Gram negatives (Gram positives: 59% vs 21%, respectively, p=0.025)

The prevalence of previous colonization, before the day of VAP diagnosis, was also analyzed. In day before VAP diagnosis the prevalence of colonization in patients that went on to developed VAP was 76.9%. However, in the previous days, from 5 to 2 days before VAP, the rate of colonization was lower (28.6%, 25%, 50%, 72.7%, respectively)

The concordance between the bacteria isolated at the day of VAP and previous colonization was 81.3%.

*Assessment of biomarkers, inflammatory and clinical variables at the day of VAP diagnosis*

At day of VAP diagnosis, all studied variables were significantly higher in VAP patients (table 3 ESM). Of the studied biomarkers and inflammatory variables, CRP was the one that performed best, with an ROC-AUC of 0.77, followed by temperature. The AUC of PCT, MR-proADM and WCC were all ≤0.68. The combination of CRP and PCT at the day of VAP diagnosis increased slightly VAP diagnostic performance (ROC-AUC=0.83). The CPIS presented also a very good performance for VAP diagnosis with an AUC of 0.92. The combination of CPIS with either CRP or PCT increased marginally the ROC-AUC to 0.95 and 0.94, respectively.

For each individual variable that were considered potentially contributive for diagnosis, those with an ROC-AUC above 0.75, we also assessed the best cut-off values. For CRP, it was 9.7 mg/dl (sensitivity 0.86, specificity 0.60, positive likelihood ratio 2.2 and negative likelihood ratio 0.2). For CPIS, it was 6 (sensitivity 0.56, specificity 0.97, positive likelihood ratio 19 and negative likelihood ratio 0.5). This means that CRP is a good biomarker to exclude VAP diagnosis, whereas CPIS is a good score to confirm.

Among VAP patients, we found no differences in biomarker levels, namely CRP, PCT and MR-proADM, when comparing early and late VAP episodes (p=0.906, p=0.446, p=0.196, respectively).

**References**

1. The problem of sepsis. An expert report of the European Society of Intensive Care Medicine. Intensive Care Med 1994;20(4):300-4.

2. Ugarte H, Silva E, Mercan D, De Mendonca A, Vincent JL. Procalcitonin used as a marker of infection in the intensive care unit. Crit Care Med. 1999;27(3):498-504.

3. Sierra R, Rello J, Bailen MA, Benitez E, Gordillo A, Leon C, et al. C-reactive protein used as an early indicator of infection in patients with systemic inflammatory response syndrome. Intensive Care Med 2004;30(11):2038-45.

4. Povoa P, Coelho L, Almeida E, Fernandes A, Mealha R, Moreira P, et al. C-reactive protein as a marker of infection in critically ill patients. Clin Microbiol infect 2005;11(2):101-8.

5. Levy MM, Fink MP, Marshall JC, Abraham E, Angus D, Cook D, et al. 2001 SCCM/ESICM/ACCP/ATS/SIS International Sepsis Definitions Conference. Crit Care Med. 2003;31(4):1250-6.

6. Garner JS, Jarvis WR, Emori TG, Horan TC, Hughes JM. CDC definitions for nosocomial infections. In: Olmsted RN, editor. APIC Infection Control and Applied Epidemiology: Principles and Practice. St. Louis: Mosby; 1996. p. A1-A20.

7. American College of Chest Physicians/Society of Critical Care Medicine Consensus Conference: definitions for sepsis and organ failure and guidelines for the use of innovative therapies in sepsis [see comments]. Crit Care Med. 1992;20(6):864-74.

8. Ewig S, Bauer T, Torres A. The pulmonary physician in critical care * 4: Nosocomial pneumonia. Thorax. 2002;57(4):366-71.

9. Heyland D, Dodek P, Muscedere J, Day A. A randomized trial of diagnostic techniques for ventilator-associated pneumonia. N Engl J Med. 2006;355(25):2619-30.

10. Mandell LA, Wunderink RG, Anzueto A, Bartlett JG, Campbell GD, Dean NC, et al. Infectious Diseases Society of America/American Thoracic Society consensus guidelines on the management of community-acquired pneumonia in adults. Clin Infect Dis 2007;44 Suppl 2:S27-72.

11. American Thoracic S, Infectious Diseases Society of A. Guidelines for the management of adults with hospital-acquired, ventilator-associated, and healthcare-associated pneumonia. Am J Respir Crit Care Med 2005;171(4):388-416.

12. Cook D, Mandell L. Endotracheal aspiration in the diagnosis of ventilator-associated pneumonia. Chest 2000;117(4 Suppl 2):195S-7S.

13. Veber B, Souweine B, Gachot B, Chevret S, Bedos JP, Decre D, et al. Comparison of direct examination of three types of bronchoscopy specimens used to diagnose nosocomial pneumonia. Crit Care Med 2000;28(4):962-8.

14. Povoa P, Teixeira-Pinto AM, Carneiro AH, Portuguese Community-Acquired Sepsis Study Group S. C-reactive protein, an early marker of community-acquired sepsis resolution: a multi-center prospective observational study. Crit Care 2011;15(4):R169.

15. Pfister R, Schwarz K, Carson R, Jancyzk M. Easy methods for extracting individual regression slopes: Comparing SPSS, R, and Excel. Tutorials in Quantitative Methods for Psychology 2013;9(2):72-8.

Table 1 ESM – Microbiology of ventilator-associated pneumonia; total isolates and the isolates in early and late ventilator-associated pneumonia episodes

| Microorganism | Total (N) | Early (N) | Late (N) |
| --- | --- | --- | --- |
| Gram positive |  |  |  |
| MSSA | 11 | 9 | 2 |
| MRSA | 4 | 2 | 2 |
| *S pneumoniae* | 1 | 1 |  |
| *Streptococcus viridans group* | 1 | 1 |  |
| Gram negative |  |  |  |
| *E coli* | 4 | 3 | 1 |
| *Enterobacter* sp. | 1 |  | 1 |
| *Klebsiella* sp. | 4 | 2 | 2 |
| *Proteus* sp. | 2 |  | 2 |
| *Citrobacter* sp. | 2 | 1 | 1 |
| *Pseudomonas aeruginosa* | 2 | 1 | 1 |
| *Acinetobacter* sp. | 1 |  | 1 |
| *Haemophilus* sp. | 6 | 2 | 4 |
| Enterobacteria, other | 1 |  | 1 |
| Bacilli Gram negative, other | 1 |  | 1 |
| Polymicrobial | 6 | 3 | 3 |

MSSA – methicillin-sensitive *Staphylococcus aureus*; MRSA – methicillin-resistant *Staphylococcus aureus*

Table 2 ESM – Assessment of kinetics (slopes, highest and Δ^max^) of studied variables between day 1 and day 6 of mechanical ventilation in ventilator-associated pneumonia patients and non-infected controls

|  | | VAP | no infection | P | ROC |
| --- | --- | --- | --- | --- | --- |
|  | | N=35 | N=70 |  |  |
| Slopes | CRP, mg/dL | 1.8 (0.6-2.6) | 0.4 (-0.7-1.2) | 0.001 | 0.71 |
|  | CRP-ratio | 0.6 (0.1-2.0) | -0.1 (-0.1-0.2) | <0.001 | 0.75 |
|  | PCT, μg/L | -0.3 (-0.6--0.2) | -0.2 (-0.5--0.2) | 0.072 | 0.61 |
|  | ADM, nmol/L | -0.1 (-0.2-0.0) | 0.0 (-0.2-0.1) | 0.814 | 0.52 |
|  | WCC, x10^3^/mm^3^ | -0.4 (-0.8-0.0) | -0,4 (-0.7-0.0) | 0.857 | 0.51 |
|  | temperature, ˚C | 0.1 (0.0-0.2) | 0.1 (0.0-0.2) | 0.43 | 0.55 |
| Highest | CRP, mg/dL | 13.8 (9.1-24.6) | 11.2 (7.9-16.9) | 0.166 | 0.59 |
|  | CRP-ratio | 4.7 (2.1-11.0) | 1.3 (1.0-2.3) | <0.001 | 0.75 |
|  | PCT, μg/L | 1.1 (0.4-9.1) | 0.4 (0.2-1.5) | 0.066 | 0.63 |
|  | ADM, nmol/L | 1.7 (1.3-3.1) | 1.3 (0.8-2.0) | 0.014 | 0.66 |
|  | WCC, x10^3^/mm^3^ | 1.1 (0.4-9.1) | 0.4 (0.2-1.5) | 0.066 | 0.63 |
|  | temperature, ˚C | 37.8 (37.2-38.5) | 37.3 (36.8-37.8) | 0.027 | 0.65 |
| Δ^max^ | CRP, mg/dL | 6.7 (3.7-13.6) | 3.2 (0.4-6.1) | <0.001 | 0.75 |
|  | CRP-ratio | 3.0 (1.1-5.3) | 0.4 (0.0-1.1) | <0.001 | 0.82 |
|  | PCT, μg/L | 0.0 (0.0-1.8) | 0.1 (0.0-0.3) | 0.774 | 0.48 |
|  | ADM, nmol/L | 0.3 (0.2-1.1) | 0.1 (-0.1-0.4) | 0.01 | 0.67 |
|  | WCC, x10^3^/mm^3^ | 2.0 (0.4-5.2) | 1.5 (0.5-3.5) | 0.619 | 0.53 |
|  | temperature, ˚C | 1.0 (0.6-1.3) | 0.8 (0.4-1.5) | 0.425 | 0.55 |

Median - 25-75th percentile; simplified CPIS – clinical pulmonary infection score; CRP – C-reactive protein; MR-proADM – mid-region fragment of pro-adrenomedullin; PCT – procalcitonin; ROC – receiver operating characteristics; VAP – ventilator associated pneumonia; WCC – white cell count;

Table 3 ESM – Biomarkers, inflammatory variables and CPIS in ventilator-associated pneumonia diagnosis (VAP and controls); values expressed in mean ± standard deviation or median [interquartile range]

|  | VAP (N=35) | Controls (N=70) | p | AUC | 95% CI |
| --- | --- | --- | --- | --- | --- |
| CRP, mg/dL | 18.1±8.4 | 10.1±6.9 | <0.001 | 0.77 | 0.67-0.88 |
| PCT, μg/L | 0.72 [3.58] | 0.31 [0.62] | 0.025 | 0.67 | 0.53-0.81 |
| ADM, nmol/L | 1.54 [2.02] | 1.08 [1.38] | 0.047 | 0.64 | 0.51-0.77 |
| WCC, x10^3^/mm^3^ | 13.2±6.3 | 9.8±3.1 | 0.005 | 0.68 | 0.55-0.81 |
| temperature, ˚C | 37.8±0.8 | 37.1±0.7 | <0.001 | 0.75 | 0.64-0.86 |
| CPIS | 6.6±1.8 | 2.6±2.1 | <0.001 | 0.92 | 0.86-0.98 |

CPIS – clinical pulmonary infection score; CRP – C-reactive protein; MR-proADM – mid-region fragment of pro-adrenomedullin; PCT – procalcitonin; VAP – ventilator associated pneumonia; WCC – white cell count.
