# Supplementary figures and images for: Biomarker kinetics in the prediction of VAP diagnosis: results from the BioVAP study
Source: Ann Intensive Care. 2016 Apr 14;6:32. doi: 10.1186/s13613-016-0134-8 (PMC4830786; doi:10.1186/s13613-016-0134-8)

Figure S1


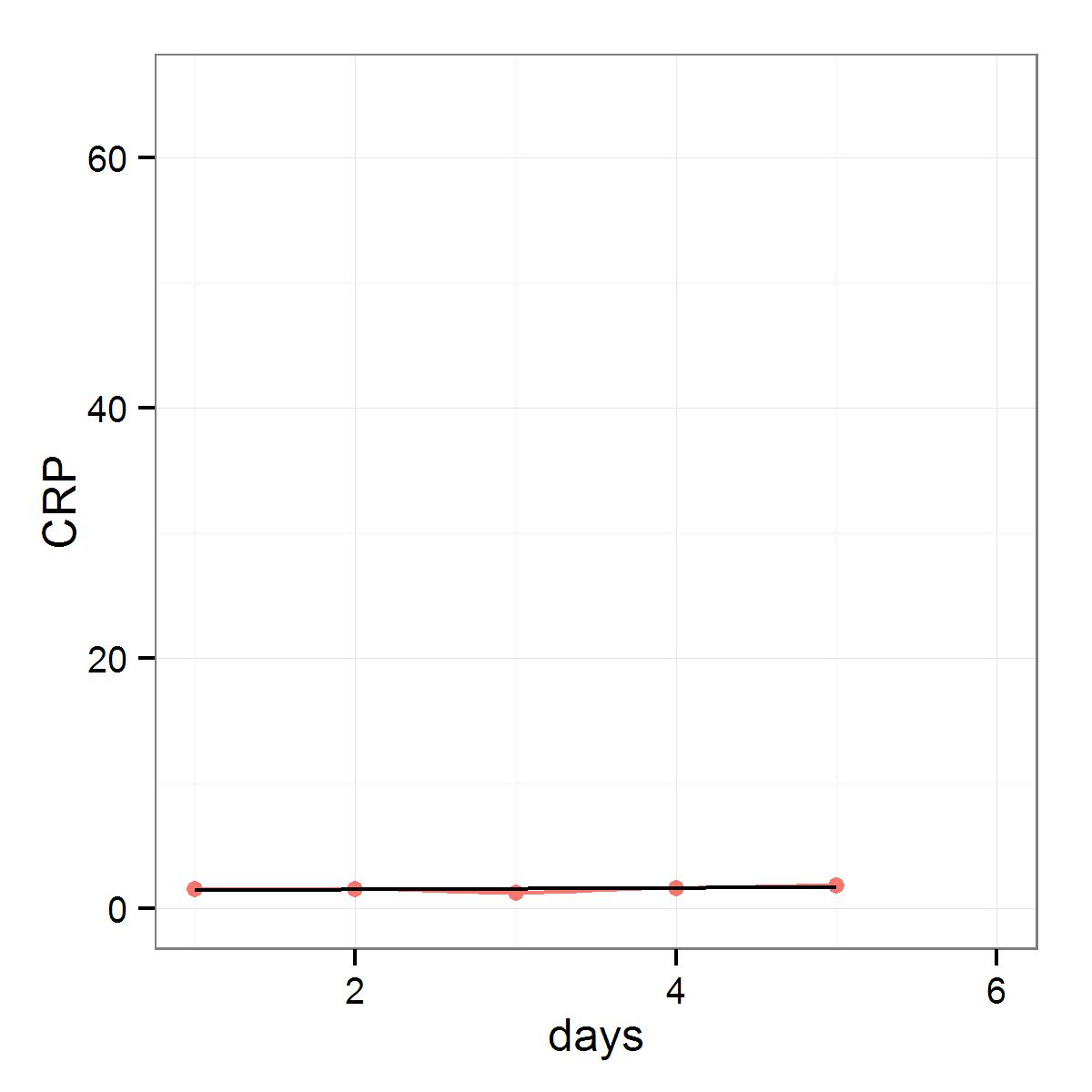

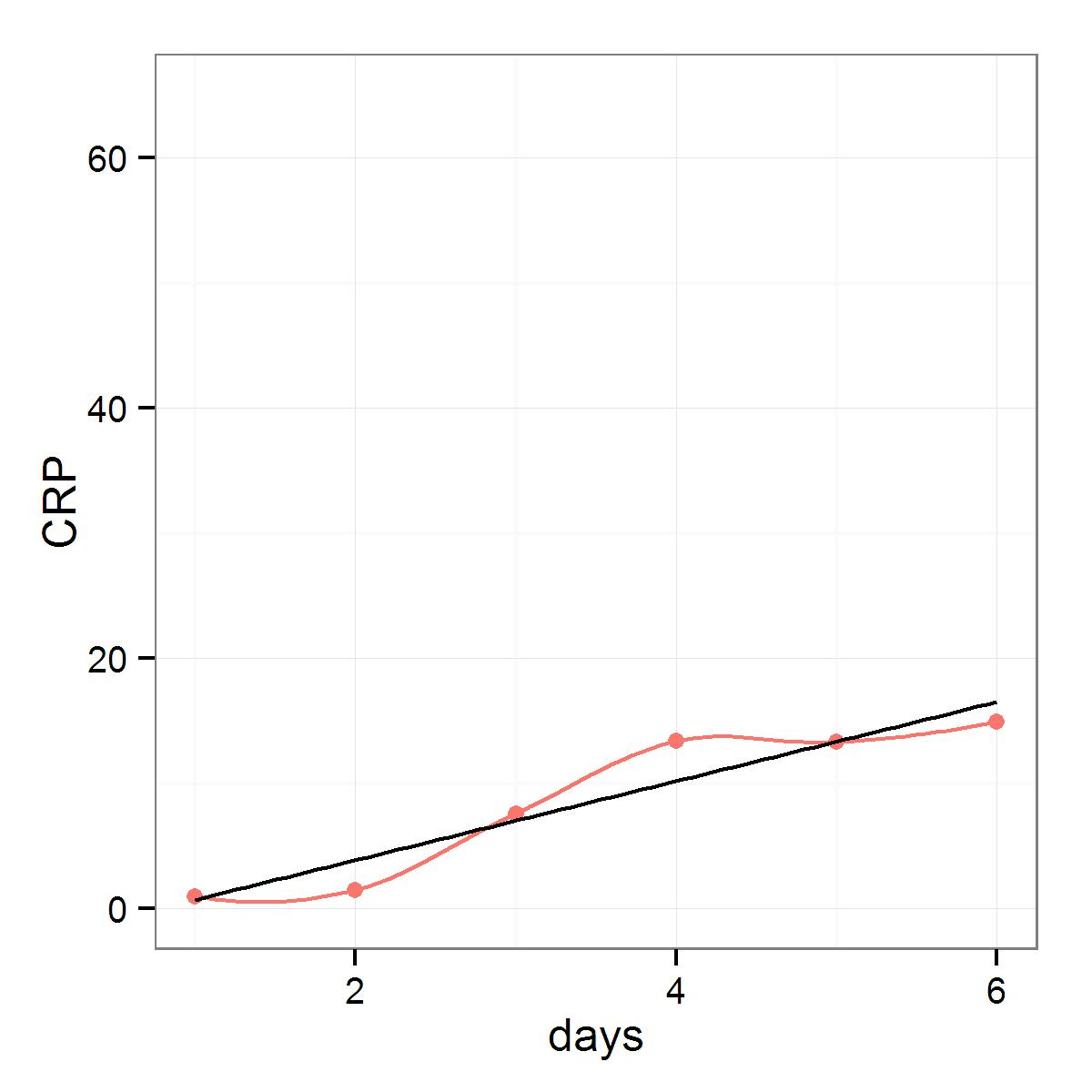

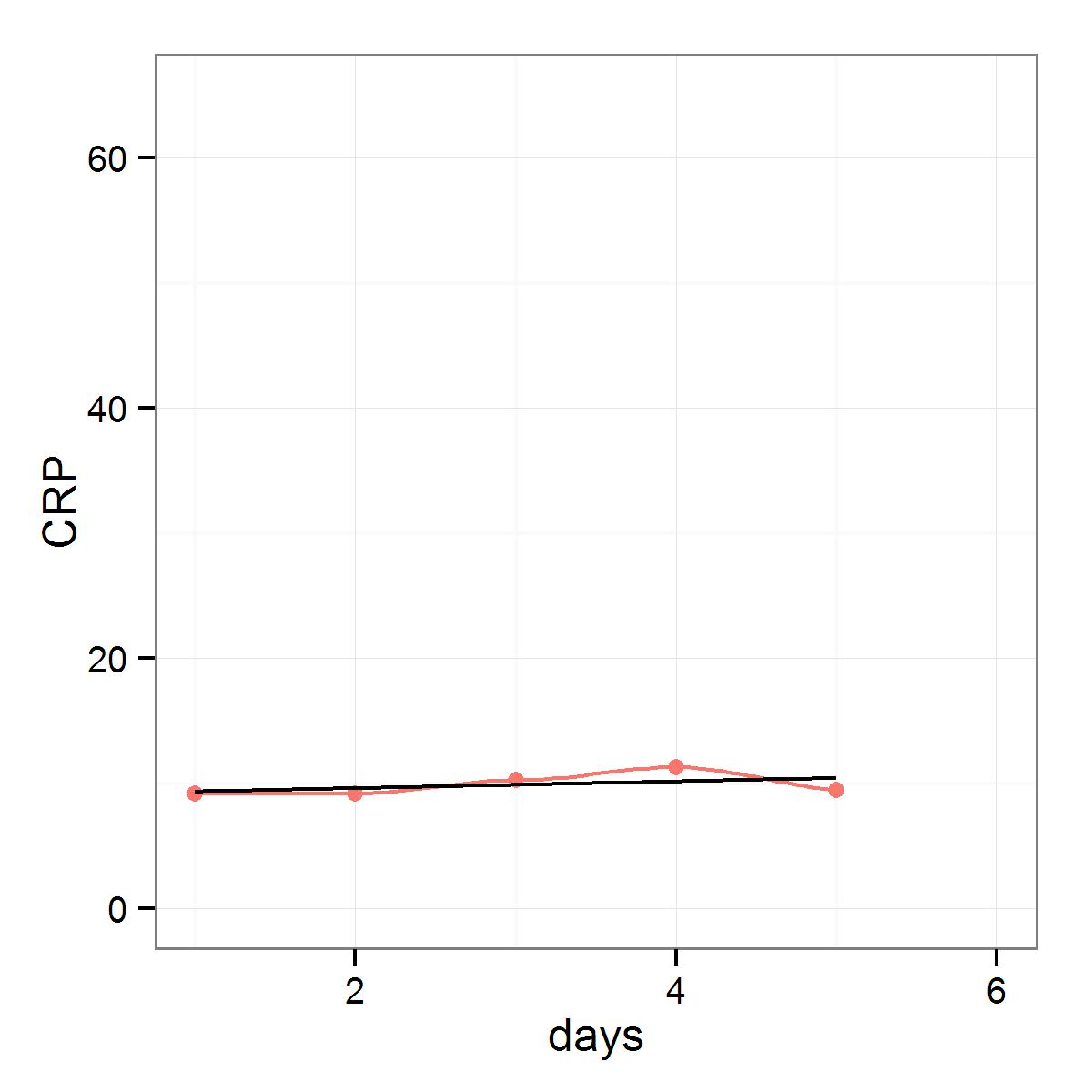


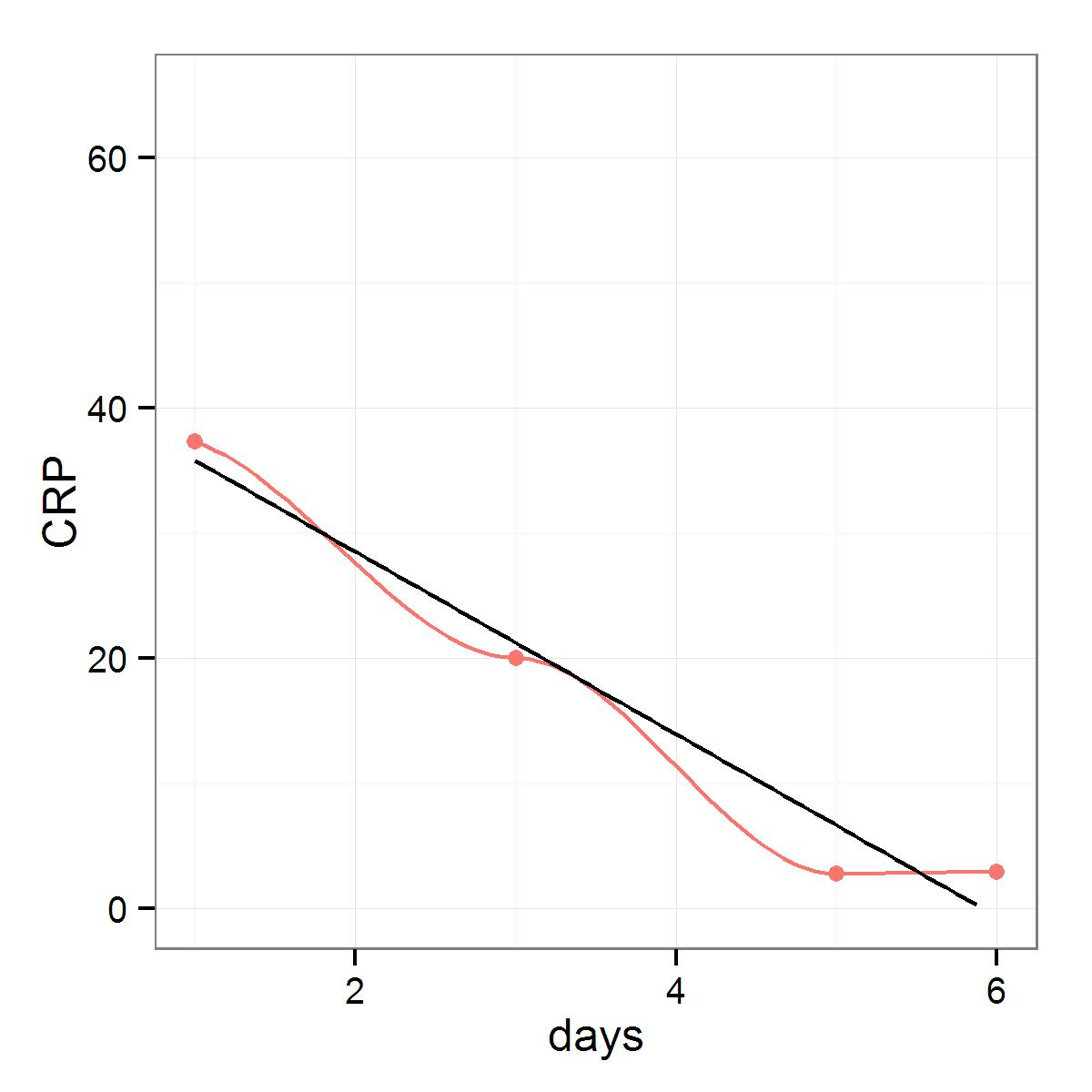

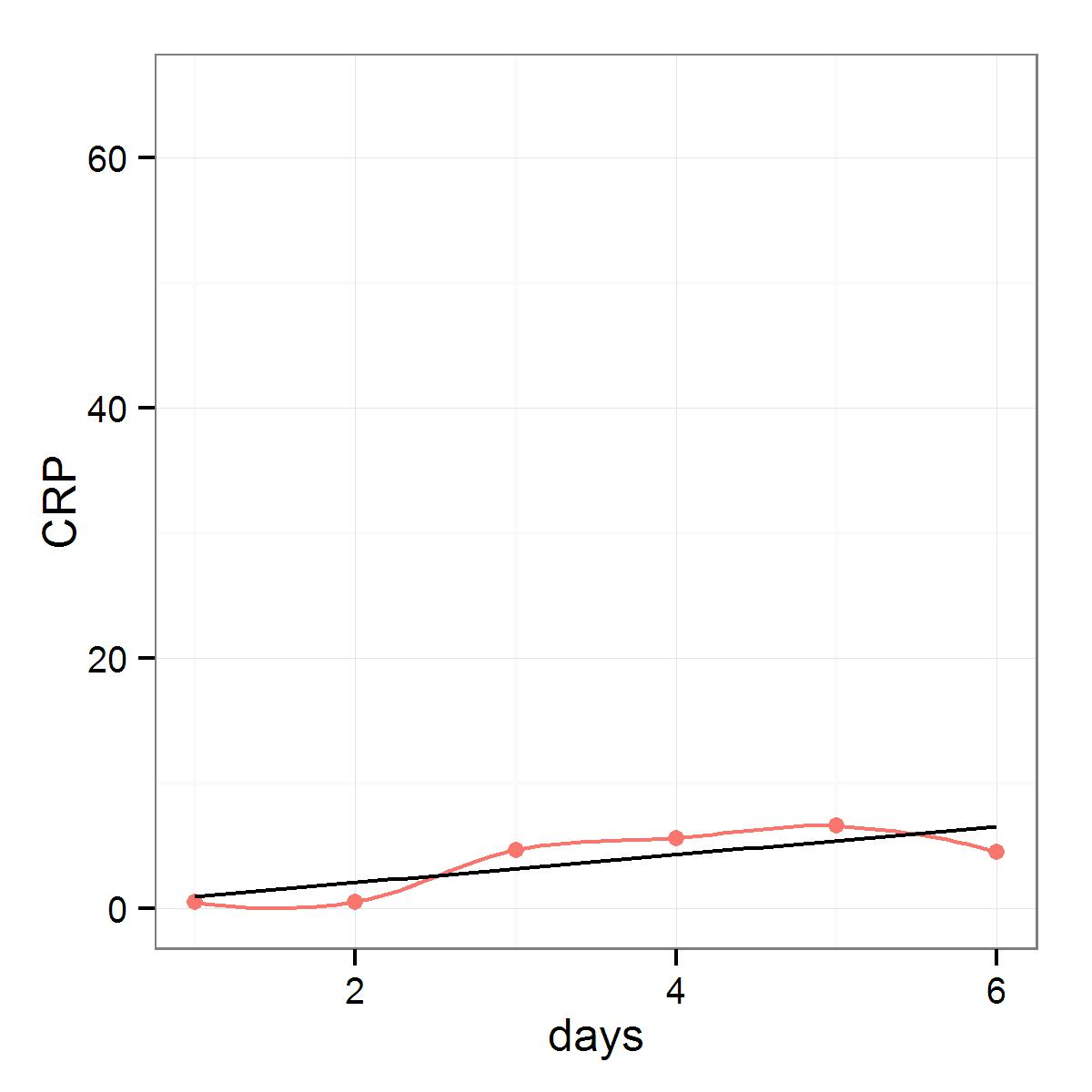

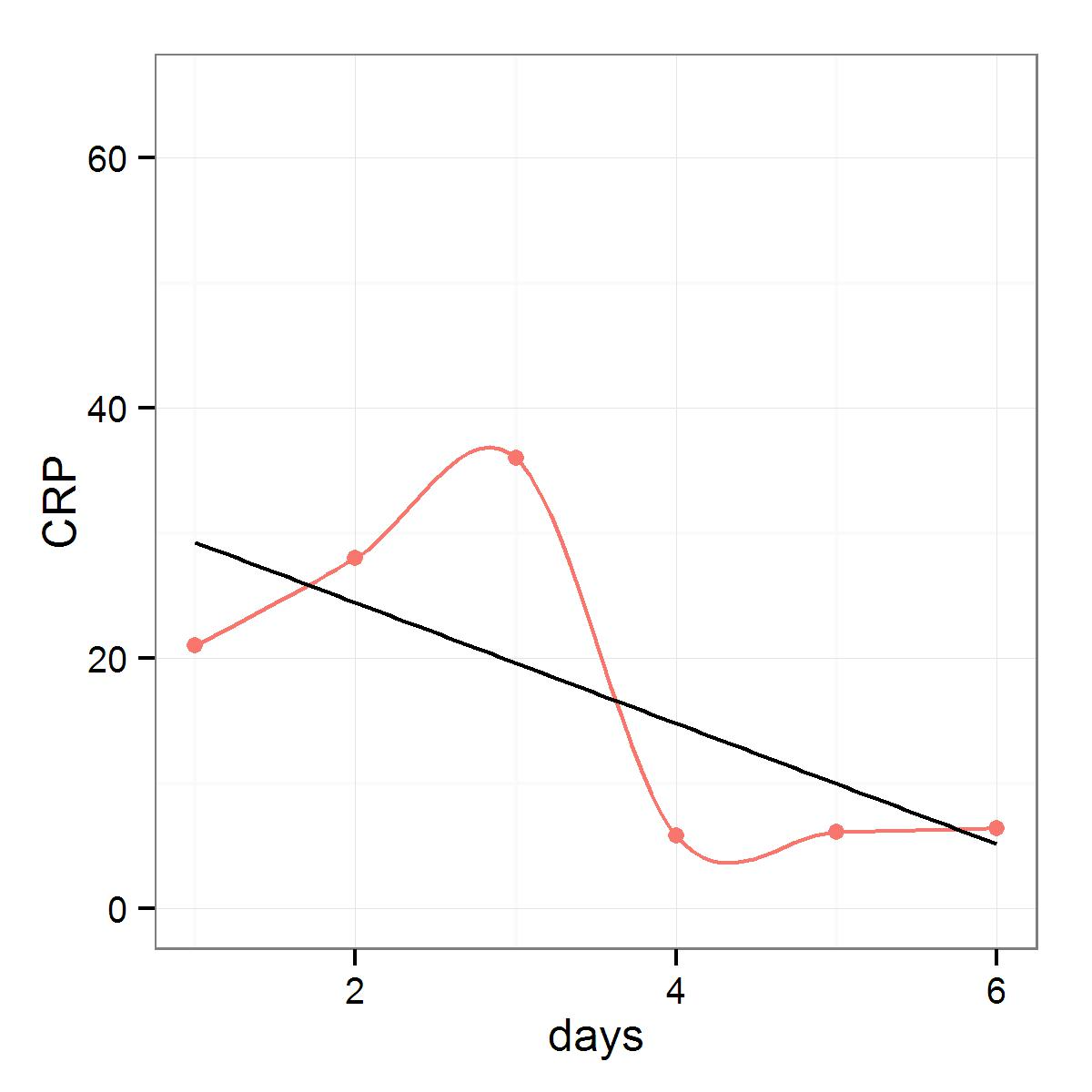


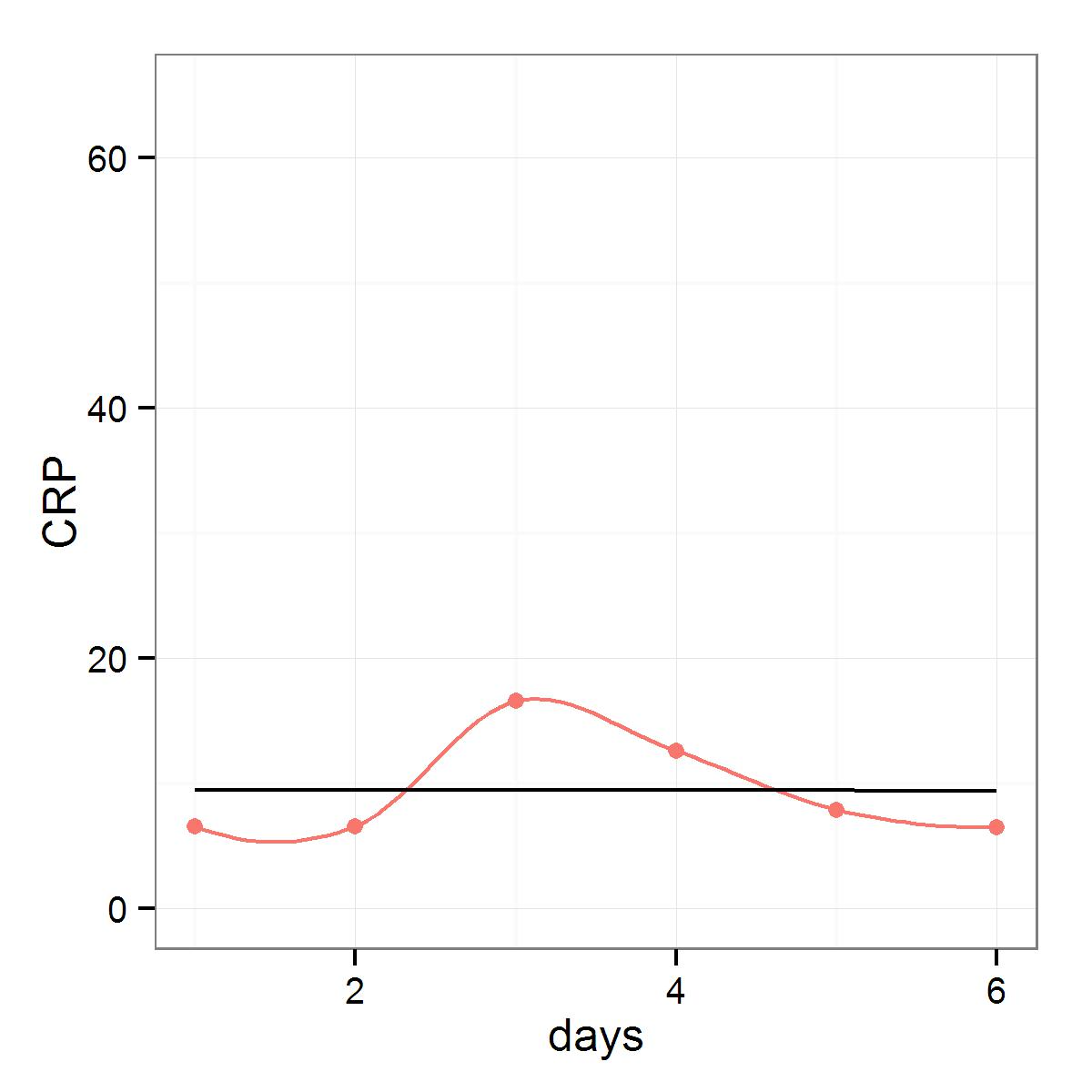

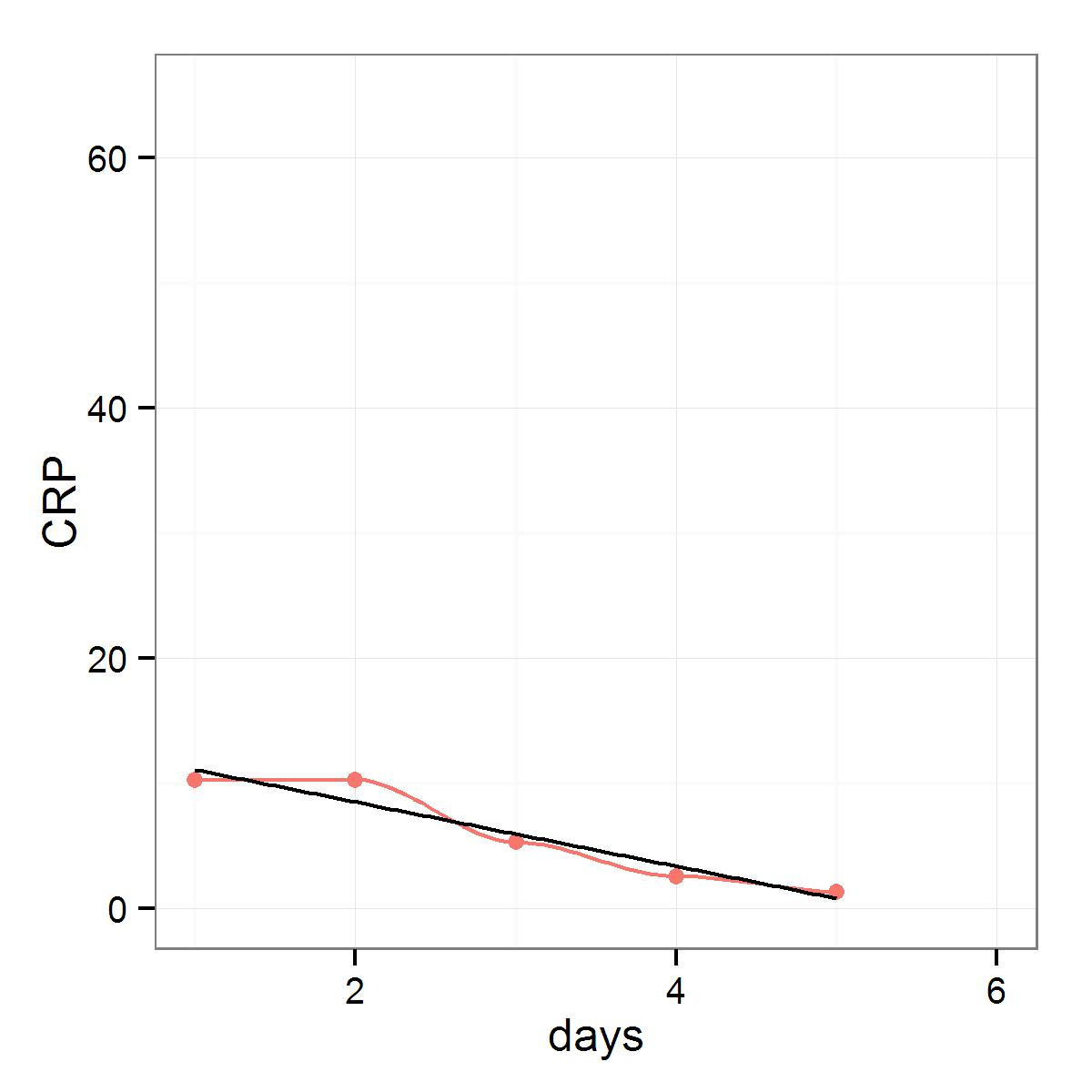

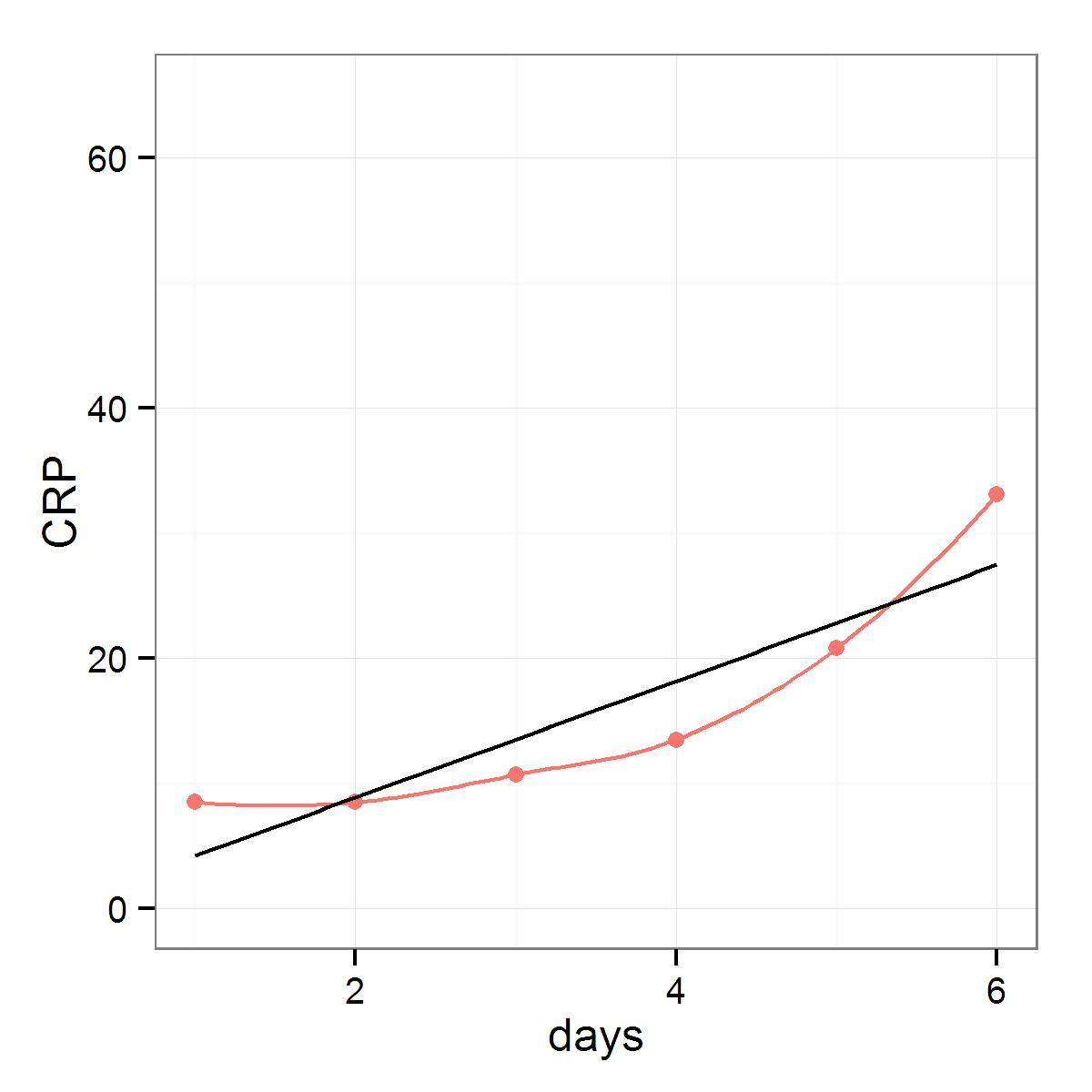


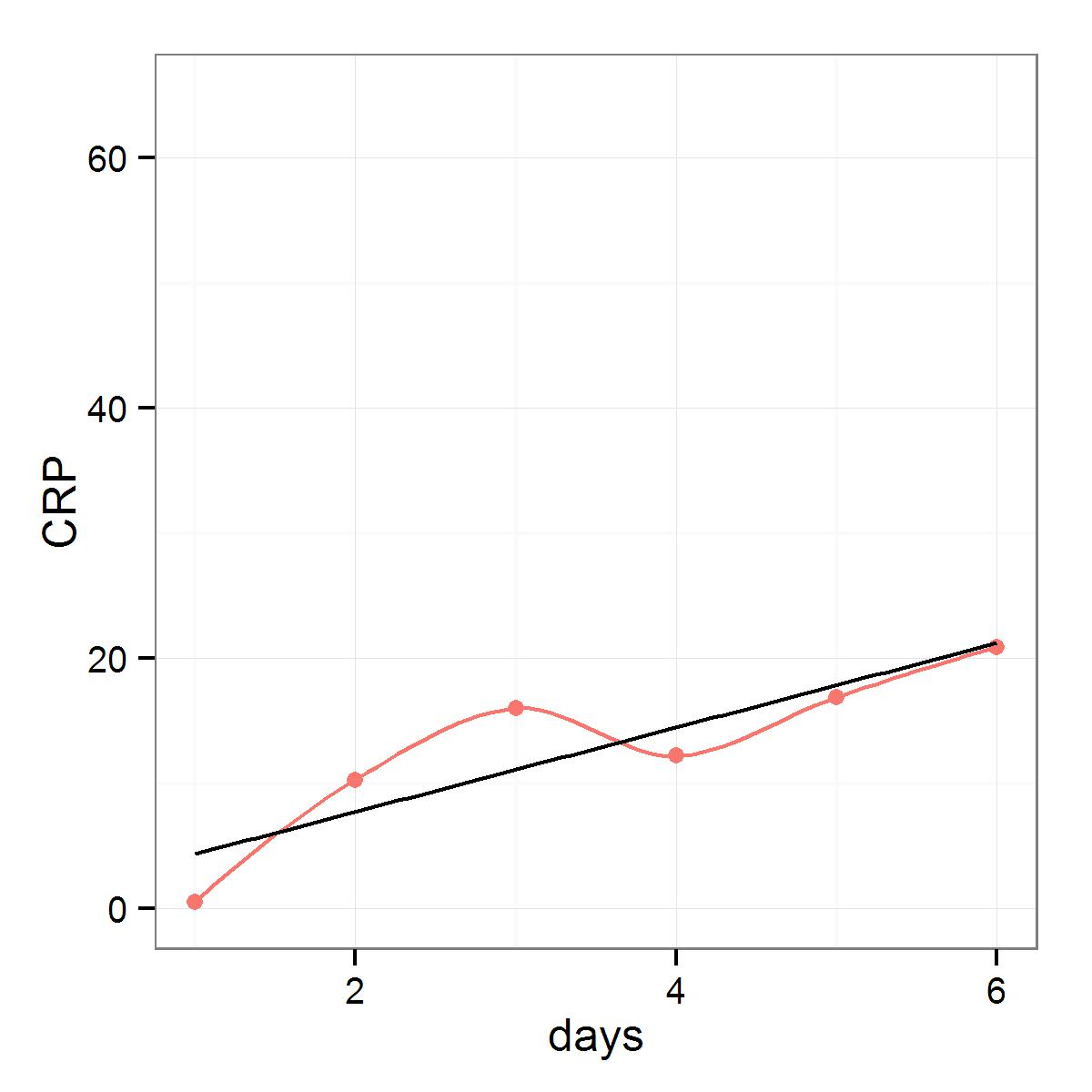

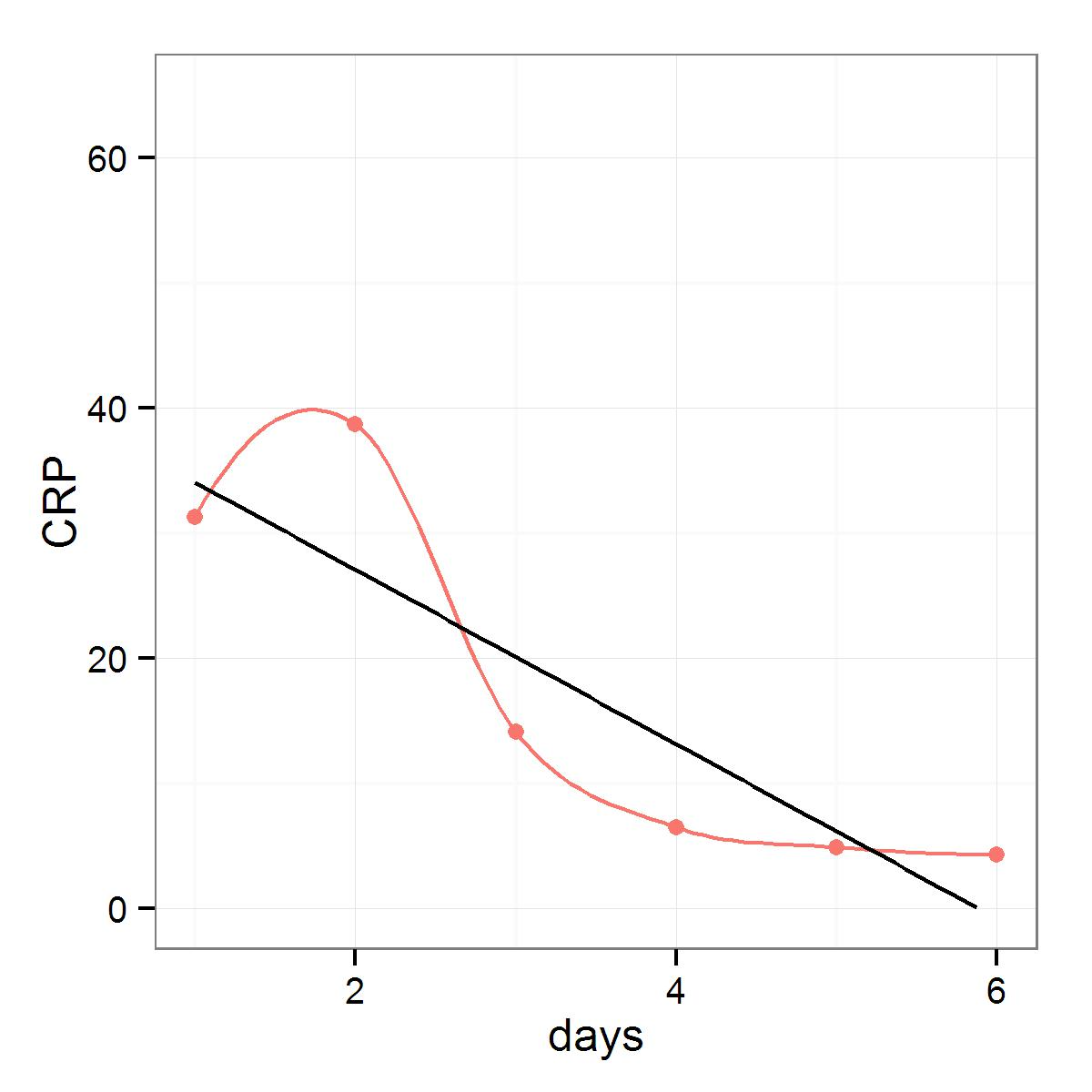

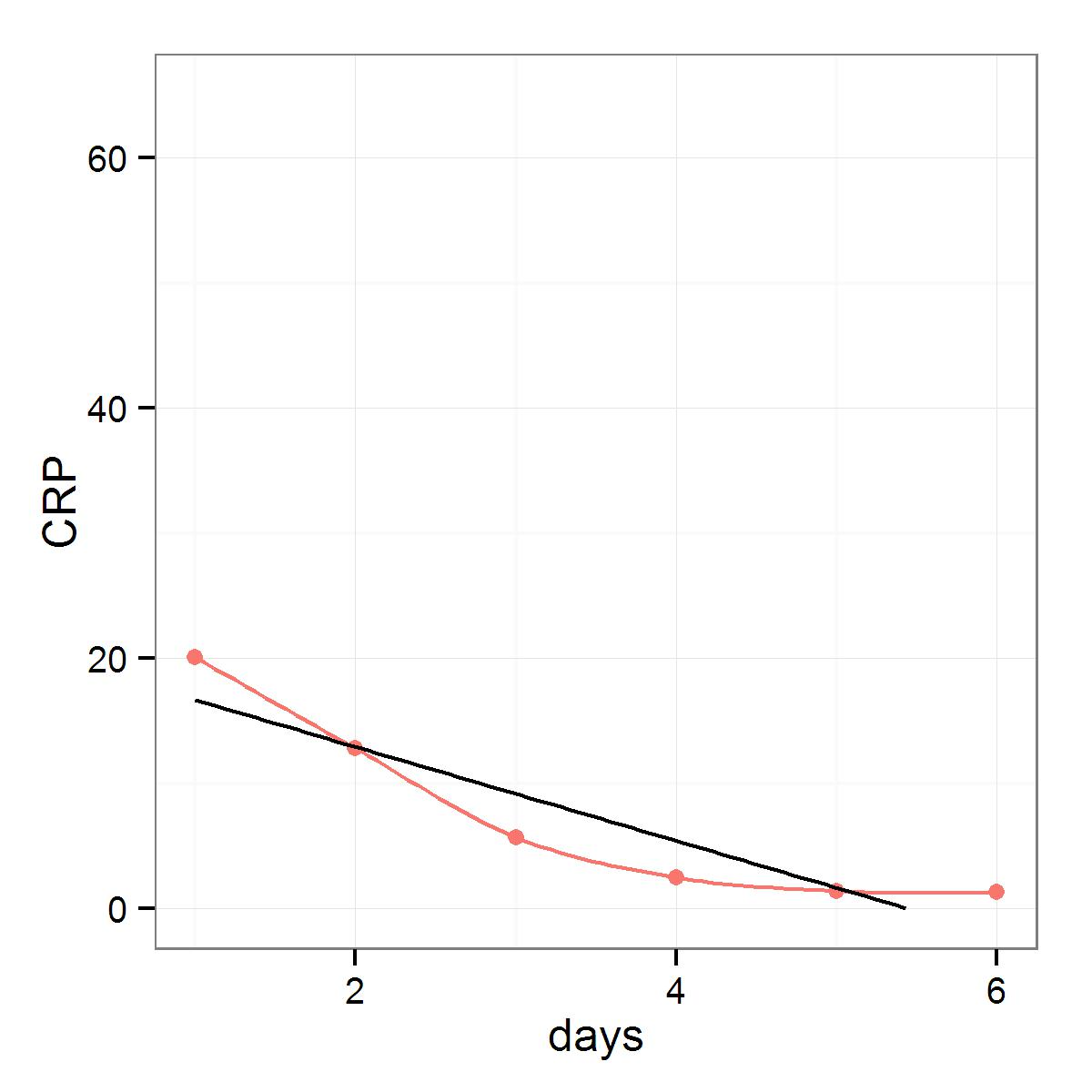

Supplement: Supplementary file 2 — 10.1186/s13613-016-0134-8 Observed values of CRP from day 1 to day 6 of mechanical ventilation and the predicted slopes by the model for a randomly selected group of patients [file 13613_2016_134_MOESM2_ESM.doc]
